# The Paralogue of the Intrinsically Disordered Nuclear Protein 1 Has a Nuclear Localization Sequence that Binds to Human Importin α3

**DOI:** 10.3390/ijms21197428

**Published:** 2020-10-08

**Authors:** José L. Neira, Bruno Rizzuti, Ana Jiménez-Alesanco, Olga Abián, Adrián Velázquez-Campoy, Juan L. Iovanna

**Affiliations:** 1 IDIBE, Universidad Miguel Hernández, 03202 Elche (Alicante), Spain; 2 Instituto de Biocomputación y Física de Sistemas Complejos, Joint Units IQFR-CSIC-BIFI, and GBsC-CSIC-BIFI, Universidad de Zaragoza, 50009 Zaragoza, Spain; ajimenez@bifi.es (A.J.-A.); oabifra@unizar.es (O.A.); adrianvc@unizar.es (A.V.-C.); 3 CNR-NANOTEC, Licryl-UOS Cosenza and CEMIF.Cal, Department of Physics, University of Calabria, Via P. Bucci, Cubo 31 C, Arcavacata di Rende, 87036 Cosenza, Italy; bruno.rizzuti@cnr.it; 4 Instituto de Investigación Sanitaria Aragón (IIS Aragón), 50009 Zaragoza, Spain; 5 Centro de Investigación Biomédica en Red en el Área Temática de Enfermedades Hepáticas y Digestivas (CIBERehd), 28029 Madrid, Spain; 6 Departamento de Bioquímica y Biología Molecular y Celular, Universidad de Zaragoza, 50009 Zaragoza, Spain; 7 Instituto Aragonés de Ciencias de la Salud (IACS), 50009 Zaragoza, Spain; 8 Fundacion ARAID, Gobierno de Aragon, 50009 Zaragoza, Spain; 9 Centre de Recherche en Cancérologie de Marseille (CRCM), INSERM U1068, CNRS UMR 7258, Aix-Marseille Université and Institut Paoli-Calmettes, Parc Scientifique et Technologique de Luminy, 163 Avenue de Luminy, 13288 Marseille, France

**Keywords:** circular dichroism, fluorescence, importin, intrinsically disordered protein (IDP), isothermal titration calorimetry (ITC), molecular docking, nuclear magnetic resonance (NMR), paralogue, peptide

## Abstract

Numerous carrier proteins intervene in protein transport from the cytoplasm to the nucleus in eukaryotic cells. One of those is importin α, with several human isoforms; among them, importin α3 (Impα3) features a particularly high flexibility. The protein NUPR1L is an intrinsically disordered protein (IDP), evolved as a paralogue of nuclear protein 1 (NUPR1), which is involved in chromatin remodeling and DNA repair. It is predicted that NUPR1L has a nuclear localization sequence (NLS) from residues Arg51 to Gln74, in order to allow for nuclear translocation. We studied in this work the ability of intact NUPR1L to bind Impα3 and its depleted species, ∆Impα3, without the importin binding domain (IBB), using fluorescence, isothermal titration calorimetry (ITC), circular dichroism (CD), nuclear magnetic resonance (NMR), and molecular docking techniques. Furthermore, the binding of the peptide matching the isolated NLS region of NUPR1L (NLS-NUPR1L) was also studied using the same methods. Our results show that NUPR1L was bound to Imp α3 with a low micromolar affinity (~5 μM). Furthermore, a similar affinity value was observed for the binding of NLS-NUPR1L. These findings indicate that the NLS region, which was unfolded in isolation in solution, was essentially responsible for the binding of NUPR1L to both importin species. This result was also confirmed by our in silico modeling. The binding reaction of NLS-NUPR1L to ∆Impα3 showed a larger affinity (i.e., lower dissociation constant) compared with that of Impα3, confirming that the IBB could act as an auto-inhibition region of Impα3. Taken together, our findings pinpoint the theoretical predictions of the NLS region in NUPR1L and, more importantly, suggest that this IDP relies on an importin for its nuclear translocation.

## 1. Introduction

NUPR1 (UniProtKB O60356) is an 82-residue-long (8 kDa), monomeric intrinsically disordered protein (IDP) with a large content of basic residues [1,2]. It does not have a stable secondary and tertiary structure, as also happens, at least partially, for other IDPs [3,4,5]. NUPR1 is involved in chromatin remodeling and transcription, and it is an important element in cell cycle regulation and cell stress response [6,7]. It is also implicated in apoptosis, forming a complex with another IDP, prothymosin α [8,9], as well as being involved in DNA binding and repair [10,11], and in the interaction with Polycomb group proteins [12]. Expression of the *NUPR1* gene is down-regulated by the presence of NUPR1L, a 97-residue-long paralogue of NUPR1; in turn, the expression of NUPR1L is p53-regulated [13]. We have recently shown that NUPR1L is also an IDP, but it has a higher tendency to self-associate than NUPR1 [14], and it shows regions with conformations including turn- or helix-like structures.

The active transport of proteins from the cytoplasm to the nucleus occurs through several transport receptors known as importins (or karyopherins), co-operating with other proteins such as GTPase Ran and nucleoporins [15,16,17]. The classical nuclear import pathway is initiated by recognition of a typical amino acid sequence (NLS, nuclear location sequence) in the cargo by an importin α [18]. The complex cargo-importin α binds to importin β, through the importin β-binding domain (IBB), and the ternary complex moves through the nuclear pore complex (NPC). The complex within the nucleus is dissociated by the action of GTPase Ran interacting with importin β, and both importins α and β are recycled back to the cytoplasm [18]. There are seven isoforms of importin α in humans, which have a role in cell differentiation, gene regulation, and cancer development [19,20]. We have chosen Impα3 as a target for NUPR1 because of its larger flexibility in comparison with other importins, as concluded from X-ray data, which confer it a greater ability to interact with different cargos. In addition, from a practical point of view, Impα3 can be also easily expressed and purified for in vitro structural studies. Interestingly, it has also been shown to be crucial in pain pathways [21].

Importin α is formed by two domains: (i) an N-terminal 60-residue-long IBB domain and (ii) a C-terminal NLS-binding motif formed by ten armadillo (ARM) repeat units [15,17,19,20]. The interaction with the cargo occurs in a concave site of the elongated structure, involving ARM motifs 2 to 4 (major site) or 6 to 8 (minor site) for the shortest monopartite NLSs, or both sets of ARM motifs for the largest bipartite NLS regions. If Importin β is not present, the IBB domain, mimicking an NLS region, occupies the same ARM motifs involved in NLS recognition, and then it has an intramolecular auto-inhibitory role [22].

We have previously shown that NUPR1 binds to human importin α3 (Impα3), also called KPNA4 [23]. NUPR1 has an NLS region involving residues in the 60–70 s along the protein sequence, as has been shown by molecular cell biology studies [24]. In this work, we studied the interaction of Impα3, and that of its truncated species without the IBB domain (∆Impα3), with NUPR1L and with its predicted NLS region, NLS-NUPR1L (comprising residues Arg51 to Gln74), using several biophysical techniques, namely, fluorescence, circular dichroism (CD), isothermal titration calorimetry (ITC), nuclear magnetic resonance (NMR) and molecular docking. Our results show that, as occurs with the parent NUPR1 [23], the intact NUPR1L was capable of interacting with both importin species with affinity in the low micromolar range (~5 μM). The NLS-NUPR1L was disordered in solution when it was in isolation, but it was bound to both importin species with similar affinity as the intact NUPR1L, suggesting that this protein region contains all the key residues determining the binding. In all cases, the affinity for ∆Impα3 was larger than for intact importin, indicating that the IBB has an auto-inhibitory effect for binding any cargo.

## 2. Results

### 2.1. Intact NUPR1L is Associated with Both Impα3 and ∆Impα3

**Figure 1:**
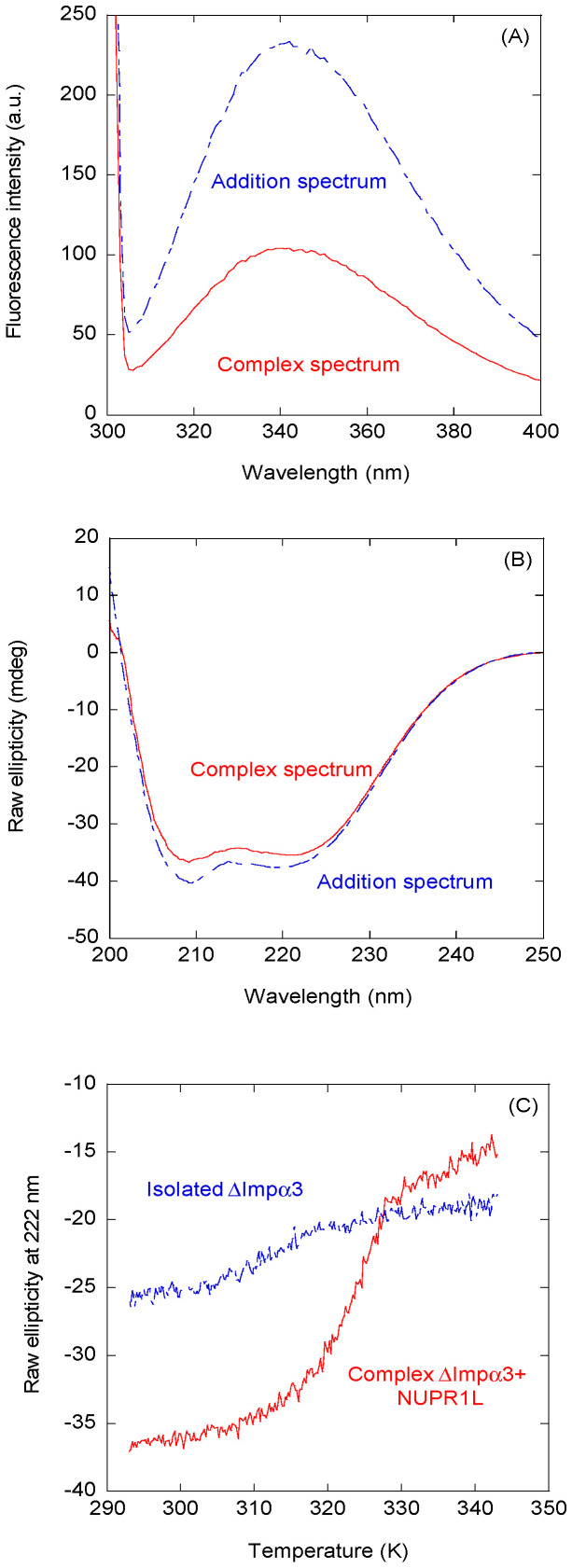
Binding of intact nuclear protein 1 NUPR1L to ∆Impα3 monitored by spectroscopic techniques: (**A**) Fluorescence spectrum obtained by excitation at 295 nm of the complex between ∆Impα3 and intact NUPR1L, and addition spectrum obtained by the sum of the spectra of both isolated macromolecules. (**B**) Far-UV circular dichroism (CD) spectrum of the complex between ∆Impα3 and NUPR1L and the addition spectrum obtained by the sum of the spectra of both isolated macromolecules. (**C**) Thermal denaturations of ∆Impα3 in the presence and absence of NUPR1L followed by the changes in ellipticity at 222 nm. All experiments were carried out in phosphate buffer (50 mM, pH 7.0).

We first determined whether intact NUPR1L could bind to Impα3 and ∆Impα3, keeping in mind that this IDP has a high tendency to aggregate [14]. We first mapped, by fluorescence and CD, whether there was binding between NUPR1L and each importin species, by comparing changes in the fluorescence and far-UV CD spectra of the complex with those obtained from the sum of the corresponding spectra of each molecule. Although the far-UV CD spectra are dominated by the presence of importins, because of their larger size and higher number of peptide bonds (when compared with that of isolated NUPR1L), the spectra can provide valuable information. Figure 1 shows the spectra obtained with ∆Impα3 (the results for Impα3 are provided in the Supplementary Material, Figure S1). The results of CD and fluorescence for ∆Impα3 indicate the following: (i) there were changes in the environment around tryptophan residues of at least one of the two proteins (i.e., NUPR1L or ∆Impα3) upon binding (fluorescence spectra, Figure 1A), and (ii) there were changes in the secondary structure of at least one of the proteins upon binding (CD spectra; Figure 1B). As NUPR1L has no well-defined structure [14], and ∆Impα3 is a large protein with a rigid, well-formed helical fold [25], we suggest that the changes in CD spectra were due to the acquisition of structure by NUPR1L. We did not attempt to deconvolute the spectrum of the complex because of the presence of two polypeptide chains, and the fact that we do not know the exact conformation of NUPR1L. We further carried out thermal denaturations followed by CD; as can be observed (Figure 1C), the apparent thermal midpoint of the unfolding of ∆Impα3 changed from 317 K to 323 K, indicating the presence of binding (leading to a stabilization of the folded state of ∆Impα3). We did not follow the binding by changes in the thermal denaturation midpoint, as monitored by fluorescence, as the sigmoidal curves of both isolated importin species obtained with this technique are not as clearly defined as those from CD [25].

**Figure 2:**
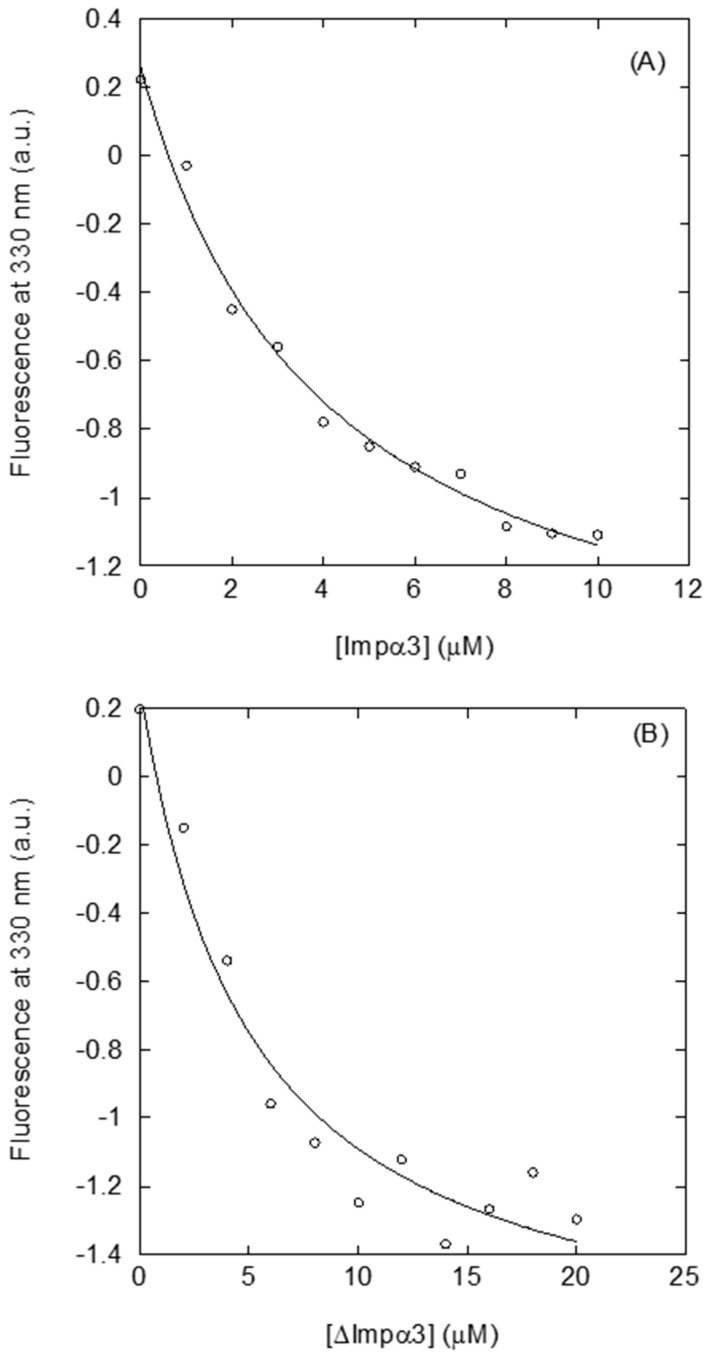
Interaction of intact NUPR1L with both importin species as measured by fluorescence: (**A**) Titration curve monitoring the changes of Impα3 fluorescence at 330 nm in the presence of NUPR1L, after excitation at 280 nm. (**B**) Titration curve monitoring the changes of ∆Impα3 fluorescence at 330 nm in the presence of NUPR1L, after excitation at 280 nm. All experiments were carried out in phosphate buffer (50 mM, pH 7.0).

Next, we tried to use ITC to determine quantitatively the binding parameters between NUPR1L and the two importins, as ITC is the gold-standard in measuring thermodynamic parameters of any binding reaction. However, a large peak observed in the thermograms upon dilution of NUPR1L precluded any measurement of the binding to the importin species contained in the cell. Then, we tried to measure the binding of NUPR1L to both importins using fluorescence (Figure 2), keeping its concentration in the cuvette constant. NUPR1L bound to both importins, with similar apparent dissociation constants, in the low micromolar range: (4.0 ± 0.7) μM for Impα3 (Figure 2A), and (5 ± 1) μM for ∆Impα3 (Figure 2B). We observed that the data for ∆Impα3 were more scattered; although we do not have a clear explanation for this finding, it might be due to the lower solubility of ∆Impα3 [25].

### 2.2. Isolated NLS-NUPR1L Was Bound to Impα3 and ∆Impα3

As intact NUPR1L associated to both importins, we wondered whether (i) the isolated predicted NLS region was capable of binding to them as well, and (ii) the affinity was the same as that of the intact protein. To that end, we first determined the conformational preferences of the isolated NLS-NUPR1L region by several spectroscopic methods.

#### 2.2.1. Isolated NLS-NUPR1L Was Monomeric and Disordered in Solution

**Figure 3:**
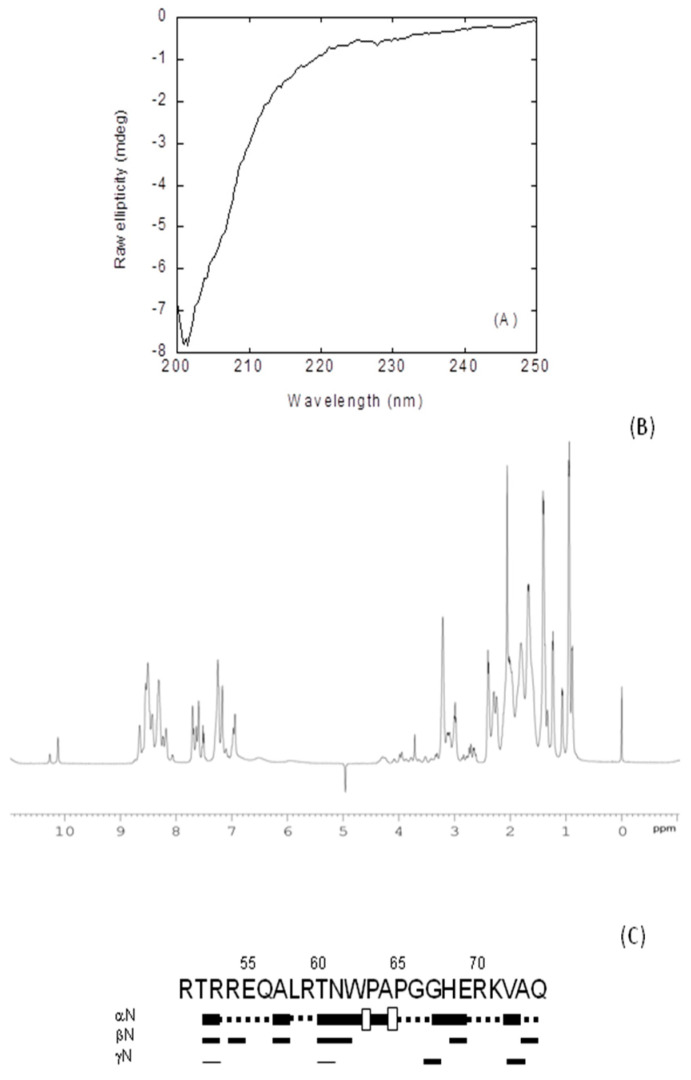
Conformational features of isolated nuclear localization sequence (NLS)-NUPR1L in solution: (**A**) Far-UV CD spectrum of NLS-NUPR1L at 298 K in phosphate buffer (50 mM, pH 7.0). (**B**) 1D-^1^H-nuclear magnetic resonance (NMR) spectrum of isolated NLS-NUPR1L at 283 K and pH 7.2 (50 mM, Tris buffer). (**C**) NOE (Nuclear Overhuaser effect) diagram of isolated NLS-NUPR1L at 283 K: NOEs are classified into strong, medium, or weak, as represented by the height of the bar underneath the sequence; signal intensity was judged by visual inspection from the NOESY (Nuclear Overhuaser effect spectrosocopy) experiments. The corresponding H_α_ NOEs with the H_δ_ of the following proline residue are indicated by an open bar in the row corresponding to the sequential αN contacts. The dotted lines indicate NOE contacts that could not be unambiguously assigned owing to signal overlap. The numbering of residues corresponds to that of the sequence of intact NUPR1L. The symbols αN, βN, γN, and NN correspond to the sequential contacts (that is, for instance, the αN corresponds to the αN (i,i + 1) contacts).

The fluorescence spectrum of the peptide had a maximum at 353 nm (Figure S2), close to the wavelength where the maximum of fluorescence for a solvent-exposed tryptophan is expected [26]; therefore, we could conclude that the sole tryptophan present in NLS-NUPR1L (Trp62) was exposed to the solvent. The CD spectrum of isolated NLS-NUPR1L did show an intense minimum at ~203 nm (Figure 3A), indicating that the peptide acquired a random-coil conformation. This was further confirmed by 1D-^1^H-NMR spectra (Figure 3B), which showed a clustering of the signals of all the amide protons between 8.0 and 8.5 ppm, and the methyl protons were observed between 0.8 and 1.0 ppm, which is a feature typical of disordered polypeptide chains [27].

The peptide was monomeric, as concluded from the value of *D* measured by the DOSY (diffusion ordered spectroscopy) and the estimated *R*_h_ obtained from comparison with that of dioxane: (1.74 ± 0.05) × 10^−6^ cm^2^ s^−1^ and (12 ± 2) Å, respectively. This value of *R*_h_ was similar to that obtained theoretically for a random-coil polypeptide [28]: 14 ± 3 Å.

To further confirm the disordered nature of NLS-NUPR1, we also carried out homonuclear 2D-^1^H-NMR experiments (Table S1). We observed NOEs between the H_α_ protons of Trp62 and the H_δ_ of Pro63, but we also observed other signals involving residues around Trp62; in fact, two signals were observed for the indole proton of Trp62 (Figure 1B) (Table S1). These results indicate the presence of the cis-trans equilibrium between the two conformations of Pro63, probably favored by the bulkiness of the side-chain of Trp62. The peptide was mainly disordered in solution, as suggested by two lines of evidence (further pinpointing the results from fluorescence (Figure S2), far-UV CD (Figure 3A), and 1D-^1^H-NMR spectra (Figure 3B). First, the sequence-corrected conformational shifts (∆δ) of H_α_ protons [27,29,30] were within the commonly accepted range for random-coil peptides (∆δ ≤ 0.1 ppm) (Table S1). Second, no long- or medium-range NOEs were detected, but only sequential ones (Figure 3C).

To sum up, all the experimental techniques concurred to indicate that the isolated NLS-NUPR1L was disordered in aqueous solution.

#### 2.2.2. Isolated NLS-NUPR1L Associated with Both Importins

In the following step, we measured the affinity of NLS-NUPR1L for both importins. We followed the same procedure as with intact NUPR1L, that is, first we tried to detect changes using fluorescence; CD; and, in this case, *T*_2_-relaxation measurements; next, we measured quantitatively the affinity using ITC and fluorescence.

Fluorescence and CD experiments showed that there were changes in the spectra upon addition of NLS-NUPR1L to each of the importin species (Figures S3 and S4), although again, the CD spectra are dominated by signal of the importins, and the effect is more marked in this case as a result of the smaller size of NLS-NUPR1L compared with the whole NUPR1L. The changes for ∆Impα3 (Figure S4) were similar (either in the steady-state spectra for both techniques or in the thermal denaturations followed by CD) to those observed for the intact NUPR1L with ∆Impα3 (Figure 1). Experiments aimed to detect binding using relaxation NMR measurements were only carried out with Impα3, because of its larger solubility [25]. It is important to note that the *T*_2_ is greater for small molecules and shorter in larger molecules (or complexes) owing to a larger number of dipole–dipole interactions [31]. The *T*_2_ of the most up-field shifted indole signal (that is, the one with the larger intensity) was measured in the isolated peptide, and was 62.2 ms; conversely, the *T*_2_ in the presence of Impα3 was 36.7 ms, in agreement with what we should expect upon complex formation [31].

**Figure 4:**
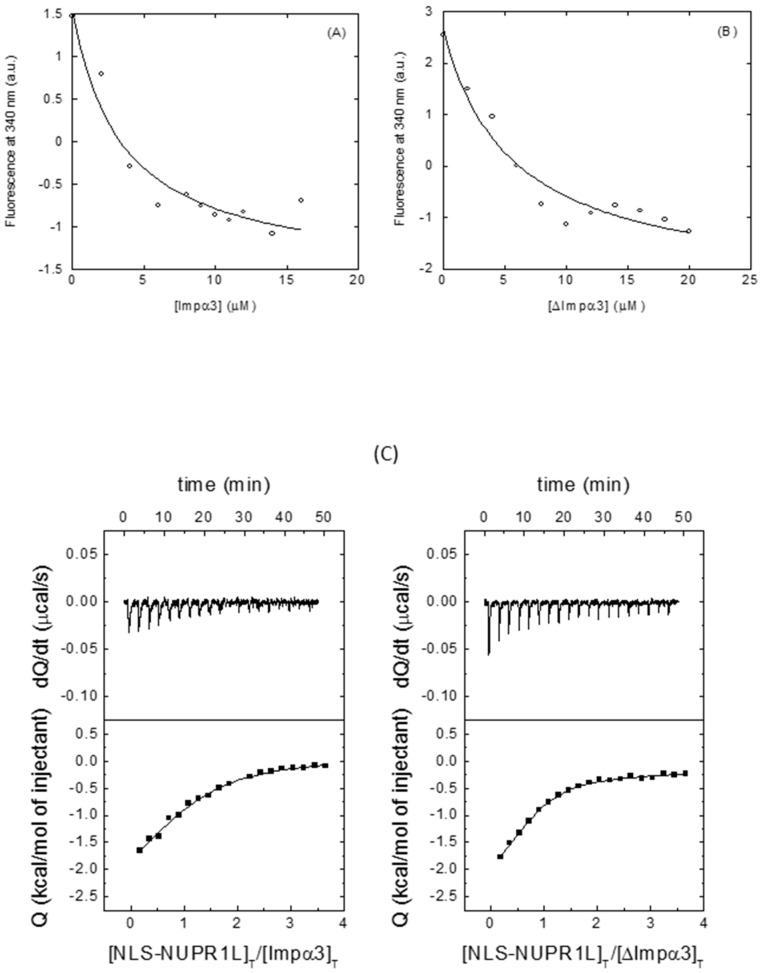
Binding of NLS-NUNPR1L to both importin species: (**A**) Titration curve monitoring the changes of Impα3 fluorescence at 340 nm in the presence of NLS-NUPR1L, after excitation at 280 nm. (**B**) Titration curve monitoring the changes of ∆Impα3 fluorescence at 340 nm in the presence of NLS-NUPR1L, after excitation at 280 nm. All experiments were carried out in phosphate buffer (50 mM, pH 7.0). (**C**) Calorimetric binding isotherms (ligand normalized heat effect per injection as a function of the ligand/protein molar ratio) for the interaction of NLS-NUPR1L with Impα3 (left) and ∆Impα3 (right) are shown, with the thermogram (raw thermal power data as a function of time) at the top of each panel. Binding parameters were estimated by non-linear least squares regression data analysis of the interaction isotherms applying a single ligand binding site model implemented in Origin 7.0. (OriginLab, Northampton, MA, USA).

Then, we proceeded to determine the binding between the same molecules using fluorescence and ITC. The fluorescence results (Table 1, Figure 4A,B) yielded values similar to those measured for the intact NUPR1L (Section 2.1), but ITC yielded a dissociation constant larger for Impα3 (12 μM), and a value similar to that measured from fluorescence for ∆Impα3 (5 μM) (Table 1, Figure 4C). Similar discrepancies in the measured affinity constants among different techniques have been observed when measuring interactions in other proteins [32,33,34,35]. The reason behind such discrepancy is related to the particular features of each technique. Steady-state techniques, where the physical observable is the equilibrium state after long incubation times that allow an optimal accommodation of the interacting molecules (such as fluorescence titration), may provide higher affinities than transient-event techniques, where the observable quantity mainly reflects the first encounter between the interacting molecules (such as ITC), thus kinetically slow readjusting conformational events may be overlooked. Because the stoichiometry of binding is already accounted for in the binding model and both importins slightly differ in the parameter n, that difference in the parameter n for both importins could be due to the lower solubility of ∆Impα3 [25], resulting in a lower fraction of active or binding-competent protein.

#### 2.2.3. Binding Regions in the Docking of NLS-NUPR1L to Importins

Molecular docking was used to predict the binding location of NLS-NUPR1L on the surface of Impα3, and to clarify the structural basis of their interactions. Because of the relatively high number of degrees of freedom of the 24-residue-long peptide used in our experiments and its large structural flexibility, our in silico research was carried out considering nine 8-residue-long fragments of this peptide, each possessing a number of rotatable bonds (ranging from 23 to 38, depending on the fragment) close to the limit considered reliable to be computationally tractable by the docking engine [36]. Any possible bias in the simulation was avoided by performing a blind docking on the whole protein volume and using a very high exhaustiveness in the search.

**Figure 5:**
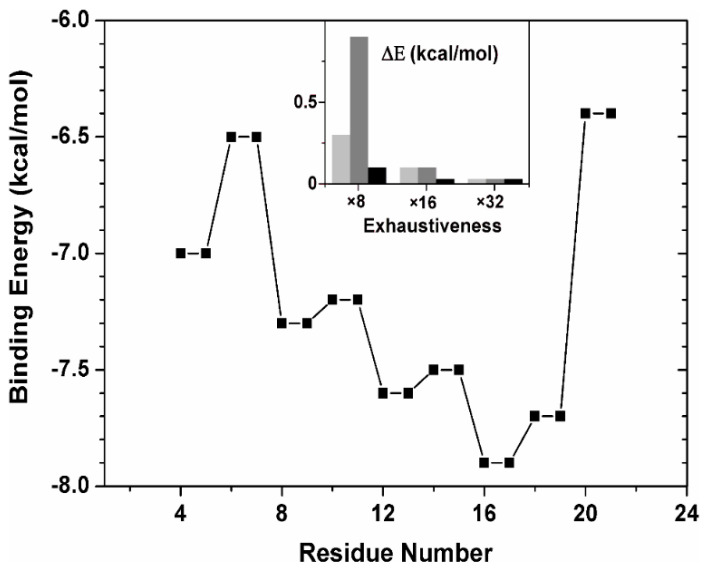
Binding energy of 8-residue-long fragments of NLS-NUPR1L peptide to importin: Affinity of each fragment is shown in correspondence of the two residues at the centre of each 8-residue sequence. (Inset) Difference in the binding energy between the docking pose with the highest affinity found at increasing exhaustiveness in the search versus the best pose found at any exhaustiveness value, for three 8-residue-long fragments that together span the whole sequence of the twenty-residue-long NLS-NUPR1L: (light grey) fragment RTRREQAL, (dark grey) RTNWPAPG, and (black) GHERKVAQ.

Figure 5 summarizes the binding affinity for ∆Impα3 of the peptide fragments, as obtained in the docking experiments. The energy value for each of the 8-residue-long fragment is reported in correspondence to its two central amino acids. We also verified that the computational depth in the docking search was reasonable to obtain a statistical convergence of the binding score values obtained (inset of Figure 5), indicating that the conformational space for the 8-residue-long fragments could be considered exhaustively sampled. The most favorable binding score was observed for the fragment with sequence PAPGGHER, which is one of the regions with the highest conformational flexibility in the parent peptide sequence due to the presence of two couples of disorder-prone Pro and Gly residues. As discussed above, this region is probably responsible for hampering the free rotation of the indole moiety of Trp62, and then of the presence of both indole signals (Figure 3B), and thus two distinct conformers. The binding energy of the fragment was −7.9 kcal/mol, indicating an affinity in the low micromolar range. The predicted core region of the NLS of NUPR1L essentially maps in correspondence with that of NUPR1 [24], although the former is shifted a few residues towards the N-terminal region of the main chain compared with the latter, when the two protein sequences are aligned. More generally, all the fragments that were part of the 14-residue-long sequence RTNWPAPGGHERKV showed energies ≤ −7.5 kcal/mol, suggesting that this whole region may contribute to the binding of NUPR1L to Impα3.

**Figure 6:**
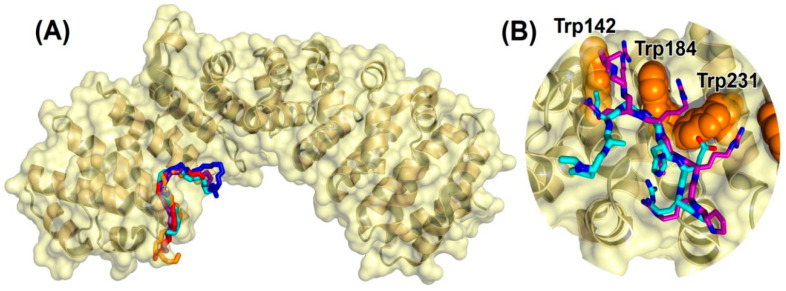
Predicted docking poses for NUPR1L peptide on importin. (**A**) Bound conformation of the capped fragment PAPGGHER: backbone (–N–C^α^–C– atoms) representation of the best five docking poses on ∆Impα3. (**B**) Most favorable binding pose of the same fragment (cyan), compared with the crystallographic conformation [37] of the NLS of the Epstein–Barr virus EBNA-LP protein (purple). For clarity, H atoms and backbone O atoms are omitted. The tryptophan residues (orange) in the major NLS-binding site of importin are labeled. All images were created with PyMol [38].

We also performed a structural analysis of the interaction between the NUPR1L sequence fragments and ∆Impα3. Figure 6A shows the best five binding modes obtained for the polypeptide fragment PAPGGHER, which includes the most favorable docking pose and other four poses with binding scores within 0.3 kcal/mol. All the fragment conformations were found to cluster in correspondence with the ARM repeats 2–4 of Impα3 (which are also present in ∆Impα3), which corresponds to the major binding site for the NLS of cargo proteins (Section 1). Moreover, as detailed in Figure 6B, the most favorable conformation of the fragment PAPGGHER was found to overlap with the NLS of the EBNA-LP protein of the Epstein–Barr virus [37], that is, the crystallographic ligand complexed with Impα3 in the protein structure used for the docking experiments. Key residues in the interaction with the NLS of NUPR1L were the tryptophans in the major binding site of Impα3 (labeled in Figure 6B), which are known to be essential in maintaining the binding with the EBNA-LP protein and in other importin–cargo complexes. A number of other amino acids of Impα3 also participated in the binding, including residue Asp192, which forms a salt bridge with the arginine residue in the peptide, Arg70, close to the C terminus of the fragment of NLS-NUPR1L. However, we cannot exclude that other electrostatic interactions occur in the intact NLS region with nearby arginines such as Arg59, Arg54, or Arg53.

## 3. Discussion

### 3.1. Identification of the NLS Region of NUPR1L

The first result of our work is that NUPR1L contains an NLS region, which is responsible for its binding to Impα3. Furthermore, the isolated region binds to both importin species with nearly the same affinity as to the whole IDP, indicating that all the key amino acids responsible for binding are mainly contained in such a polypeptide patch. Therefore, importins are capable of binding to both paralogues, NUPR1L and NUPR1, as we had already demonstrated the binding to the latter protein [23]. These findings are at variance with recent results obtained by fluorescence, where other IDPs were suggested to translocate into the nucleus without the need for the nucleus-cytoplasm transport machinery [39].

The docking simulation makes clear a number of points about the interaction between NUPR1L and Impα3. We identified a core region of the NLS of the NUPR1L, which is located in the same regions of the NLS already predicted and validated for the paralogue, NUPR1 [23,24]. Such a region of NUPR1L is a hot spot that binds to the major NLS-binding site of Impα3, located in the ARM repeats 2–4. The bound conformation of NLS-NUPR1L overlaps with the one obtained in crystallography for the NLS of the Epstein–Barr virus EBNA-LP protein [37] (Figure 6B). Hydrophobic interactions with the tryptophan residues in the binding site of Impα3 were crucial for the binding, suggesting a common cargo-binding mechanism shared by well-folded proteins and IDPs. In addition, these in silico results support our fluorescence findings, as it was possible to measure the binding between each importin and either the intact protein or the isolated peptide, by following the changes in fluorescence of both at 280 or 295 nm (Figure 1 and Figure 4A,B), indicating the tryptophans were involved in the reaction. Electrostatic interactions with the charged residue Asp192 of Impα3 provide a further anchor for Arg70 in the NLS of NUPR1L, contributing to securing its bound position the outmost C-terminal region of the NUPR1L peptide used in our experiments.

We also demonstrated that isolated NLS-NUPR1L did not have any propensity to acquire helix- or turn-like conformations; this result is important as we have previously shown that NUPR1L has a tendency to form locally folded regions around Trp62 [14], and with the present findings, we can conclude that that folded conformation around this region was not helical (Table S1). In this aspect, NLS-NUPR1L behaves not differently from any other NLS region of a well-folded protein [15,20,22,40]; that is, it is disordered both in isolation and when participating in forming the complex with importins (in our studies, the latter conclusion was obtained from our docking simulations). Finally, it is important to pinpoint that Trp62 is also involved in the binding of NUPR1L to prothymosin α [14]; therefore, it seems that this residue can be classified as a hot spot of NUPR1L in the association with other molecular partners.

### 3.2. The Inhibitory Effect of the IBB in Impα3

We can conclude (Table 1 and Section 2.2.2) that the removal of IBB from Impα3 promotes a more favorable binding of the NLS-NUPR1 to the ARM 2–3 units of importins: the dissociation constants were 5.5 μM (for ∆Impα3) versus 12 μM (for Impα3). Unfortunately, we cannot draw any defined conclusion for the intact NUPR1L, as we could not measure the binding parameters by ITC (Section 2.1). Nevertheless, the result obtained is in agreement with previous findings of other NLS regions of well-folded proteins [40] or with those of the intact NUPR1 protein (1.4 μM for Impα3) [23] or peptides comprising the NLS region of NUPR1 [41]. The presence of the IBB (which contains a large quantity of lysine amino acids) always exerts an auto-inhibitory effect, and the domain hampers the anchoring of NLS-NUPR1L into the major NLS-binding region of Impα3. The modulation of the complex formation between importins and their cargos (belonging to otherwise well-folded proteins) has been attributed to the IBB [18]; interestingly enough, this region is involved even in the formation of a homodimeric species between importins [42], conferring to this protein a reduced ability to bind cargos.

### 3.3. Binding to Impα3 of NUPR1L

As the isolated NLS regions of both NUPR1 and NUPR1L contain the key residues to attain binding to Impα3, and with the peptides, we could measure the binding by ITC, we shall focus our attention on the comparison between the affinities of the two paralogues for those measurements. Comparison of the values of Table 1 for NLS-NUPR1L with those of the NLS region of NUPR1 (1.7 μM for Impα3 and 0.95 μM for ∆Impα3 [41]) indicate that the binding is stronger in the case of NUPR1. Therefore, although both paralogues bind to the same molecules ([14] and this work), their affinity for the different partners is dissimilar. This could provide a mechanism to explain the regulation between these proteins, not only at a DNA level, but also at a post-translational stage.

In the case of the intact proteins, although we do not have the whole set of values of *K*_d_ obtained with the same technique (for NUPR1L, the dissociation constants were obtained by fluorescence (Section 2.1), whereas for NUPR1, they were obtained by ITC [23,41]), it is important to consider that NUPR1L in solution is an oligomer and, therefore, the self-association equilibrium will affect the apparent values of the dissociation constants determined from the experiments, which could vary depending on the self-association state of the protein.

## 4. Materials and Methods

### 4.1. Materials

Ampicillin and isopropyl-β-D-1-tiogalactopyranoside were from Apollo Scientific (Stockport, UK). Imidazole, kanamycin, TSP ((trimethylsilyl)-2,2,3,3-tetradeuteropropionic acid), Trizma base, and His-Select HF nickel resin were from Sigma-Aldrich (Madrid, Spain). Triton X-100 and protein marker (PAGEmark Tricolor) were from VWR (Barcelona, Spain). Amicon centrifugal devices with a cut-off molecular weight of 30 or 50 kDa were from Millipore (Barcelona, Spain). The rest of the materials were of analytical grade. Water was deionized and purified on a Millipore system.

### 4.2. Protein Expression and Purification

Expression and purification of codon-optimized, His-tagged ∆Impα3 (residues 64-521) were carried out using BL21 (DE3) cells [25,40]. The DNA of the codon-optimized, intact Impα3 was synthesized by NZYtech (Lisbon, Portugal) and cloned into the pHTP1 vector (kanamycin resistance), and with a His-tag at the protein N terminus. Expression and purification of Impα3 were carried out as those for ∆Impα3 in the same *E. coli* strain cells. The protein concentration of both species was determined from their six tyrosines and six tryptophans [43]. NUPR1L was expressed and purified as described [14], and its concentration was determined from its single tryptophan and its five tyrosines [43].

### 4.3. Prediction and Synthesis of NLS-NUPR1L

The NLS-NUPR1L peptide was synthesized by NZYtech with a purity of 95%. The NLS region of NUPR1L was predicted using the whole sequence of NUPR1L in the web server http://nls-mapper.iab.keio.ac.jp/cgi-bin/NLS_Mapper_form.cgi [44,45]. The predicted region with a larger score comprised residues Gly46 to Gln74. The peptide was designed to maximize solubility, comprising residues Arg51 to Gln74, with acetylation and amidation at the N and C termini, respectively, to avoid fraying effects.

### 4.4. Fluorescence

#### 4.4.1. Steady-State Fluorescence

Fluorescence spectra were collected on a Cary Varian spectrofluorometer (Agilent, Santa Clara, CA, USA), interfaced with a Peltier unit. All experiments were carried out at 298 K. Following the standard protocols used in our laboratories, the samples were prepared the day before and left overnight at 278 K; before experiments, samples were left for 1 h at 298 K. A 1 cm pathlength quartz cell (Hellma, Kruibeke, Belgium) was used. The concentration of NLS-NUPR1L was 10 μM and those of both importins were 4 μM. Samples containing the isolated peptide, the isolated importin species, and a mixture of both (at those indicated concentrations) were prepared. Experiments were acquired in 50 mM phosphate buffer (pH 7.0). For the experiments with intact NUPR1L, a concentration of 15 μM (in protomer units) was used and that of each importin was 5 μM.

Protein samples were excited either at 280 or 295 nm. The other experimental parameters and the buffers used have been described elsewhere [46]. Appropriate blank corrections were made in all spectra.

#### 4.4.2. Binding Experiments

For the titration between either Impα3 or ∆Impα3 with NUPR1L, increasing amounts of both importins, in the range 0–10 μM, were added to a solution with a fixed concentration of the intact IDP (8 μM). To maintain consistency, the same experimental set-up was used for titration of NLS-NUPR1L with both importins, although the peptide did not have any tendency to aggregate (Section 2.2.1); a fixed concentration of 8.5 μM of peptide was used in the titrations. Experiments were carried out in 50 mM buffer phosphate (pH 7.0) at 298 K. In all cases, the appropriate blank-corrections with the corresponding amounts of each importin species were subtracted. Spectra were corrected for inner-filter effects during fluorescence excitation [47]. Each titration (Impα3 with NUPR1L, Impα3 with NLS-NUPR1L, ∆Impα3 with NUPR1L, and ∆Impα3 with NLS-NUPR1L) was repeated at least three times, using new samples.

The samples were prepared the day before and left overnight at 278 K; before measurements, the samples were incubated for 1 h at 298 K. The dissociation constant of the corresponding complex, *K*_d_, was calculated by fitting the binding isotherm obtained by plotting the observed fluorescence change as a function of importin concentration to the general binding model explicitly considering ligand depletion [48,49]:

(1) $$F=F_{0}+\frac{\Delta F_{\max}}{2{\left[NUPR1L-polypep\right]}_{T}}\left[\begin{array}{l} \left({\left[NUPR1L-polypep\right]}_{T}+{\left[\mathrm{Imp}\alpha 3-\mathrm{species}\right]}_{T}+K_{d}\right)\\ -{\left(\begin{array}{l} {\left({\left[NUPR1L-polypep\right]}_{T}+{\left[\mathrm{Imp}\alpha 3-\mathrm{species}\right]}_{T}+K_{d}\right)}^{2}\\ -4{\left[NUPR1L-polypep\right]}_{T}{\left[\mathrm{Imp}\alpha 3-\mathrm{species}\right]}_{T} \end{array}\right)}^{1/2} \end{array}\right]$$

where *F* is the measured fluorescence at any particular concentration of Impα3 or ∆Impα3 after subtraction of the blank with the same concentration of either Impα3 or ∆Impα3; ∆*F*_max_ is the largest change in the fluorescence of NUPR1L or NLS-NUPR1L when the whole amount of each polypeptide formed the complex compared with the fluorescence of each isolated chain; *F*_0_ is the fluorescence intensity when no importin species was added; [NUPR1L-polypep]*_T_* is the constant, total concentration of either NUPR1L or NLS-NUPR1L; and [Impα3-species]*_T_* is that of either Impα3 or ∆Impα3, which was varied during the titration. Fitting to the above equation was carried out using KaleidaGraph version 3.5. (Synergy software, Reading, PA, USA).

### 4.5. CD

Far-UV CD spectra were collected on a Jasco J810 spectropolarimeter (Jasco, Tokyo, Japan) with a thermostated cell holder, and interfaced with a Peltier unit at 298 K. The instrument was periodically calibrated with (+)-10-camphorsulphonic acid. A cell with a path length of 0.1 cm was used (Hellma, Kruibeke, Belgium). All spectra were corrected by subtracting the corresponding baseline. The concentration of each polypeptide (importin species and either NLS-NUPR1L or intact NUPR1L) was the same as that used in the fluorescence experiments (Section 4.4).

#### 4.5.1. Far-UV CD Spectra

Isothermal wavelength spectra of each isolated macromolecule and that of the complex were acquired with five scans at a scan speed of 50 nm/min, a response time of 2 s, and a band-width of 1 nm. Samples were prepared the day before and left overnight at 278 K to allow for equilibration. Before starting the experiments, samples were further left for 1 h at 298 K. Experiments were carried out at 298 K in 50 mM buffer phosphate (pH 7.0).

#### 4.5.2. Thermal Denaturations

The experiments were performed at heating rates of 60 K/h and a response time of 8 s. Thermal scans were collected by following the changes in ellipticity at 222 nm typically from 298 to 343 K. The rest of the experimental set-up was the same as that reported in the steady-state experiments. No difference was observed between the scans aimed to test drifting in the signal of the spectropolarimeter. Thermal denaturations were not reversible for any of the polypeptides or their complexes, as shown by the following: (i) comparison of spectra before and after heating; and (ii) changes in the voltage of the instrument detector [50]. The apparent thermal denaturation midpoint was estimated from a two-state equilibrium equation, as previously described [46].

### 4.6. ITC

The experimental set-up and data processing of ITC experiments have been described previously [51]. Impα3 or ∆Impα3 (at 10–20 μM) was loaded into the cell of an Auto-iTC200 calorimeter (MicroCal, Malvern-Panalytical, Malvern, UK) and NLS-NUPR1L in the syringe (150–300 μM) in buffer Tris 50 mM, pH 8. The temperature for all experiments was 298 K. The experiments were analyzed applying a model considering a single ligand binding site (1:1 stoichiometry for the NLS-NUPR1L/Impα3 (or ∆Impα3) interaction) implemented in Origin 7.0 (OriginLab, Northampton, MA, USA).

### 4.7. NMR

The NMR experiments were acquired at 283 K on a Bruker Avance spectrometer (Bruker GmbH, Karlsruhe, Germany), equipped with a triple resonance probe and z-pulse field gradients. All experiments with NLS-NUPR1L were carried out at pH 7.2, 50 mM deuterated Tris buffer (not corrected for isotope effects). The spectra were calibrated with TSP ((trimethylsilyl)-2,2,3,3-tetradeuteropropionic acid), by considering pH-dependent changes of its chemical-shifts [31]; probe temperature was calibrated with methanol [31].

#### 4.7.1. D-^1^H-NMR Spectrum

An amount of 128 scans was acquired with 16 K acquisition points for the homonuclear 1D-^1^H-NMR spectrum, and using a peptide concentration of 1.0–1.2 mM. Water signal was suppressed using the WATERGATE sequence [52]. The spectrum was processed with Bruker TopSpin 2.1 (Bruker GmbH, Karlsruhe, Germany), after zero-filling and apodization with an exponential window.

#### 4.7.2. Translational Diffusion NMR (DOSY)

The peptide concentration in DOSY experiment was 120 μM, and 128 scans were acquired, where the gradient strength was varied linearly. Translational self-diffusion measurements were performed with the pulsed-gradient spin-echo sequence in the presence of 100% D_2_O. Experimental details have been described elsewhere [46]. The gradient strength was varied in sixteen linear steps between 2 and 95% of the total power of the gradient coil. The gradient strength was calibrated using the value of the translational diffusion coefficient, *D*, for the residual proton water signal in a sample containing 100% D_2_O in a 5 mm tube [53]. The length of the gradient was 2.25 ms, the time between the two pulse gradients in the pulse sequence was 200 ms, and the recovery delay between the bipolar gradients was 100 μs. The methyl groups with signals between 1.0 and 0.80 ppm were used for peak integration (Section 2.2.1). Fitting of the exponential curves, obtained from experimental data as previously described [46], was carried out with KaleidaGraph version 3.5 (Synergy Software, Reading, PA, USA). A final concentration of 1% of dioxane, which was assumed to have a hydrodynamic radius *R*_h_ = 2.12 Å [53], was added to the peptide solution.

#### 4.7.3. D-^1^H-NMR Spectroscopy

Two-dimensional spectra were acquired in each dimension in the phase-sensitive mode using the time-proportional-phase incrementation technique (TPPI) and a spectral width of 7801.69 Hz [54]; the final concentration of the NLS-NUPR1L was the same as that used in the 1D experiments. Standard TOCSY (Total correlation spectroscopy) (with a mixing time of 80 ms) [55] and NOESY experiments (with a mixing time of 250 ms) [56] were performed by acquiring a data matrix size of 4096 × 512 points. The DIPSI (decoupling in the presence of scalar interactions) spin-lock sequence [57] was used in the TOCSY experiments with 1 s of relaxation time. Typically, 96 scans were acquired per increment in the first dimension, and the residual water signal was removed using the WATERGATE sequence [52]. NOESY spectra were collected typically with 96 scans per increment in the first dimension, with the residual water signal removed again by the WATERGATE sequence [52], and with 1 s of relaxation time. Data were zero-filled, resolution-enhanced with a square sine-bell window function optimized in each spectrum, baseline-corrected, and processed with the Bruker TopSpin 2.1 software (Bruker GmbH, Karlsruhe, Germany). The ^1^H resonances were assigned by standard sequential assignment processes [27]. The chemical shift values of H_α_ protons in random-coil regions were obtained from tabulated data, corrected by neighbouring residue effects [27,29,30].

#### 4.7.4. Measurements of *T*_2_

Measurements of the *T*_2_ (transverse relaxation time) provide a convenient method to determine the molecular mass of a macromolecule, as the correlation time, τ_c_, is approximately equal to 1/(5 × *T*_2_) [58]. We measured the *T*_2_ of one of the indole protons (Section 2.2.1) for NLS-NUPR1L (at 35 μM concentration) in isolation and in the presence of Impα3 (at a final concentration of 7 μM) with the 1-1 echo sequence [59]. The calculation of the *T*_2_ was carried out as described [58].

### 4.8. Molecular Docking

Molecular simulations of the interaction between the NLS-NUPR1L and Impα3 were performed using AutoDock Vina 1.1.2 [36], on the basis of a protocol already used to screen the binding of the NLS and other fragments of the parent protein NUPR1 [12,60]. The structure of ∆Impα3 (without the IBB) was modelled starting from entry 5X8N of the Protein Data Bank (PDB), in which the intact monomeric protein is crystallized in complex with the NLS of the EBNA-LP protein of the Epstein–Barr virus [37].

The 24-residue (capped) sequence Ac-RTRREQALRTNWPAPGGHERKVAQ-NH_2_ for the NLS-NUPR1L peptide used in our experiments possesses 99 rotatable dihedral angles; therefore, its conformational space is too large to be systematically explored. To overcame this difficulty, we employed nine 8-residue fragments (RTRREQAL, RREQALRT, GHERKVAQ) spanning the whole peptide sequence and differing by a shift of two consecutive amino acids. All the fragments were capped through acetylation (CH_3_–CO–) and N-methyl amidation (–NH–CH_3_) to mimic the missing regions of the peptide main chain, except the N-terminal end of the last fragment, in which standard amidation (–NH_2_) was preserved. Docking simulations were carried out considering the whole protein surface (volume size 50 Å × 90 Å × 90 Å), and with very high exhaustiveness (up to 32 times larger than the default value) during the search [61].

## Figures and Tables

**Table 1:** Thermodynamic parameters at 298 K in the binding reaction of nuclear localization sequence (NLS)-NUPR1L to the two importin species. NUPR1, nuclear protein 1; ITC, isothermal titration calorimetry.

	Fluorescence		ITC		
Importin Species		*K*_d_ (μM)	*K*_d_ (μM)	∆*H* (kcal mol^−1^)	*n*
Impα3		3 ± 1	12 ± 2	−3.1 ± 0.5	1.04 ± 0.05
∆Impα3		5 ± 2	5.5 ± 0.9	−2.4 ± 0.5	0.75 ± 0.06

## Supplementary Materials

The following are available online at https://www.mdpi.com/1422-0067/21/19/7428/s1: There is one Table S1 containing the NMR assignment of the peptide, and four Figures in the Supplementary Material (Figures S1–S4).

## Abbreviations

ARM	Armadillo
CD	Circular dichroism
DOSY	Diffusion ordered spectroscopy
DIPSI	Decoupling in the presence of scalar interactions
IBB	Importin β-binding domain
IDP	Intrinsically disordered protein
Impα3	Human importin α3 isoform (residues 1–521)
∆Impα3	Truncated species of Impα3 (residues 64-521) depleted of the IBB
ITC	Isothermal titration calorimetry
NLS	Nuclear localization sequence
NLS-NUPR1L	Nuclear localization sequence of NUPR1L (residues 51–74)
NOE	Nuclear Overhauser effect
NOESY	Nuclear Overhauser effect spectroscopy
NPC	Nuclear pore complex
NUPR1	Nuclear protein 1
NUPR1L	The NUPR1-like paralogue
TOCSY	Total correlation spectroscopy
TPPI	Time-proportional-phase incrementation technique
UV	Ultraviolet
