# Supplementary material for: The Paralogue of the Intrinsically Disordered Nuclear Protein 1 Has a Nuclear Localization Sequence that Binds to Human Importin α3
Source: Int J Mol Sci. 2020 Oct 8;21(19):7428. doi: 10.3390/ijms21197428 (PMC7583046; doi:10.3390/ijms21197428)
Supplement: Supplementary file 1 [file ijms-21-07428-s001.pdf]

Supplementary Information for the manuscript

The paralogue of the intrinsically disordered nuclear protein 1 has a nuclear localization sequence that binds to human importin α3

José L. Neira, Bruno Rizzuti, Ana Jiménez-Alesanco, Olga Abián, Adrián Velázquez-Campoy and Juan L. Iovanna

SUPPLEMENTARY FIGURE LEGENDS

FIGURE S1: **Binding of intact NUPR1L to Impα3 monitored by spectroscopic techniques**: (A) Fluorescence spectrum obtained by excitation at 295 nm of the complex between Impα3 and intact NUPR1L, and addition spectrum obtained by the sum of the spectra of both isolated macromolecules. (B) Far-UV CD spectrum of the complex between Impα3 and NUPR1L and the addition spectrum obtained by the sum of the spectra of both isolated macromolecules. (C) Thermal denaturations of Impα3 in the presence and absence of NUPR1L followed by the changes in ellipticity at 222 nm. The data on the y-axis has been scaled up. All experiments were carried out in phosphate buffer (50 mM, pH 7.0).

FIGURE S2: **Structural features of isolated NLS-NUPR1L as monitored by fluorescence**: The spectrum of NLS-NUPR1L after excitation at 280 nm. Spectrum was acquired in phosphate buffer (50 mM, pH 7.0) at 298 K.

FIGURE S3: **Binding of NLS-NUPRL1 to ΔImpα3 as monitored by changes in the fluorescence and CD spectra**: (A) Changes occurring upon binding of NLS-NUPR1L to ΔImpα3 as monitored by far-UV CD. (B) Changes in fluorescence spectra upon addition of NLS-NUPR1L to ΔImpα3, after excitation at 280 nm. (C) Thermal denaturations followed by

ellipticity at 222 nm (far-UV CD) of isolated ΔImpα3 and its complex with NLS-NUPR1L. Experiments were acquired in in phosphate buffer (50 mM, pH 7.0) at 298 K.

FIGURE S4: **Binding of NLS-NUPRL1 to Impα3 as monitored by changes in the fluorescence and CD spectra**: (A) Changes occurring upon binding of NLS-NUPR1L to Impα3 as monitored by far-UV CD. (B) Changes in fluorescence spectra upon addition of NLS-NUPR1L to Impα3, after excitation at 280 nm. (C) Thermal denaturations followed by ellipticity at 222 nm (far-UV CD) of isolated Impα3 and its complex with NLS-NUPR1L. The data on the y-axis has been scaled up. Experiments were acquired in phosphate buffer (50 mM, pH 7.0) at 298 K.

Fig. S1 (Neira et al.)

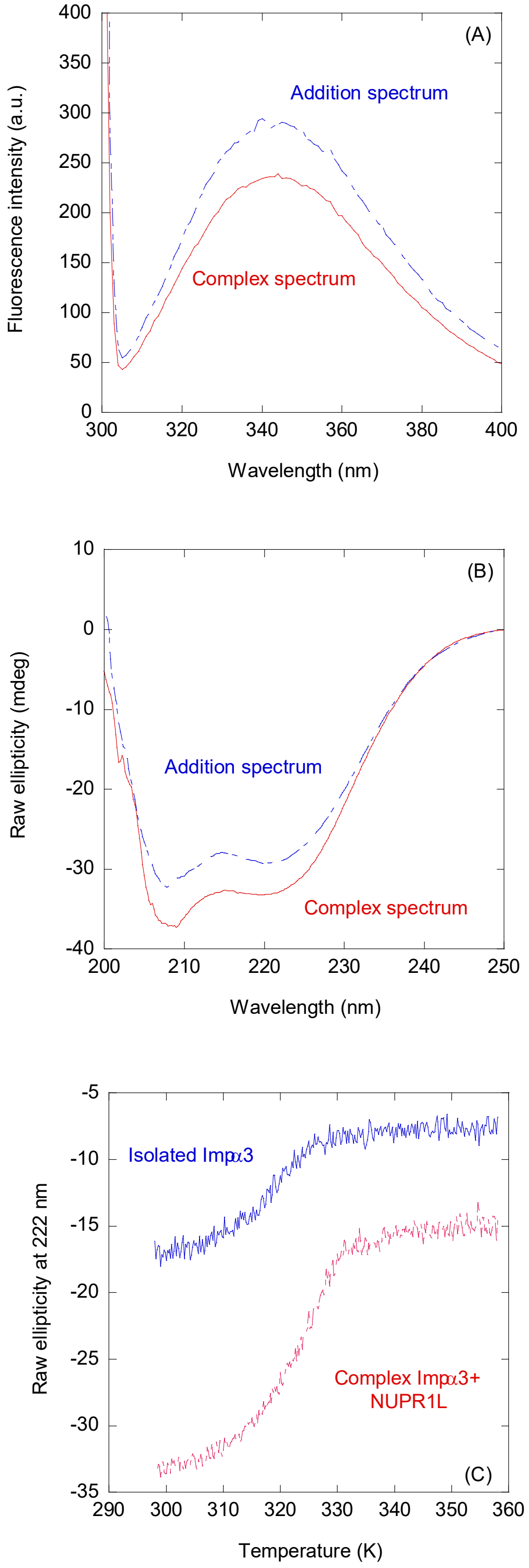

Fig. S2 (Neira et al.)

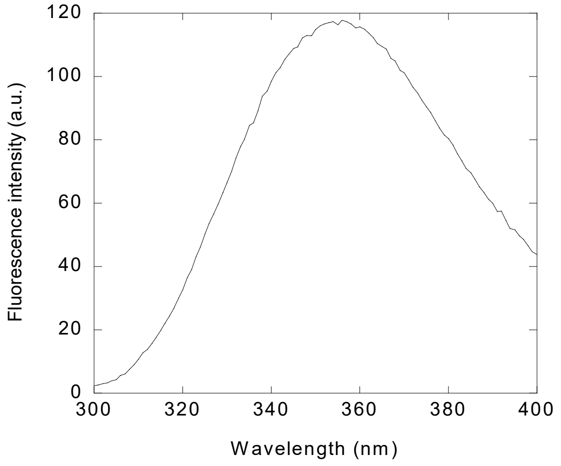

Fig. S3 (Neira et al.)

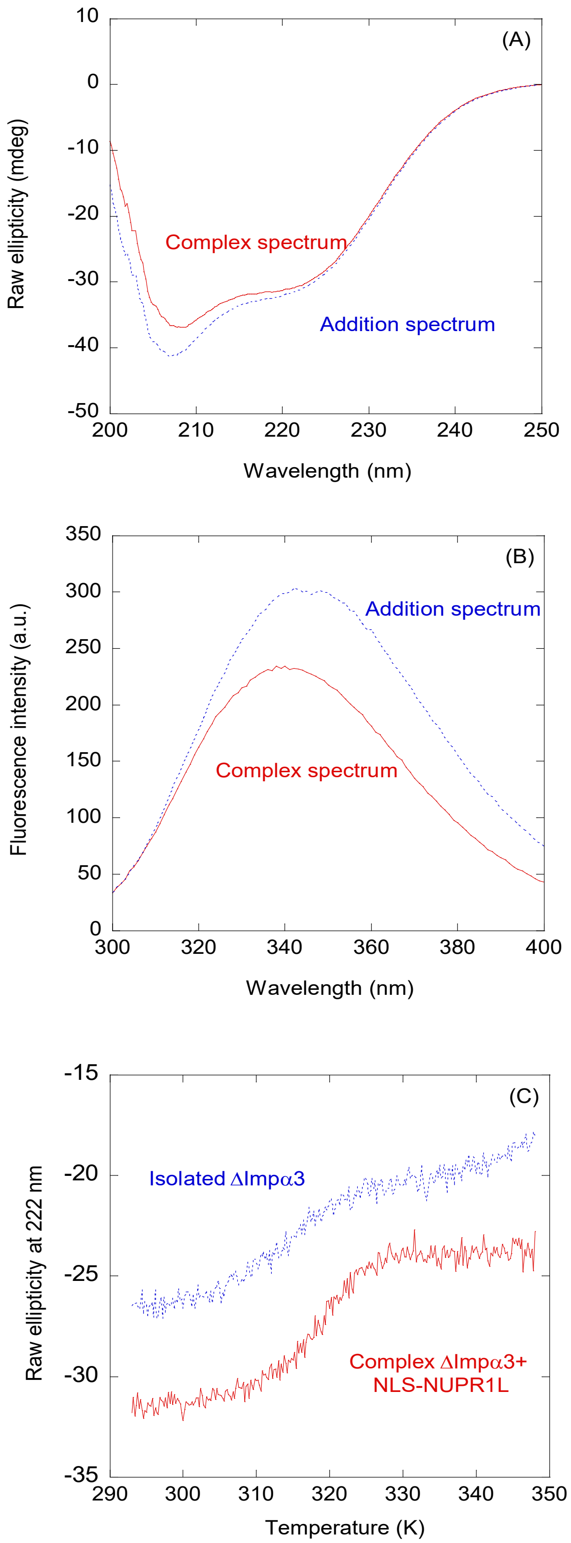

Fig. S4 (Neira et al.)

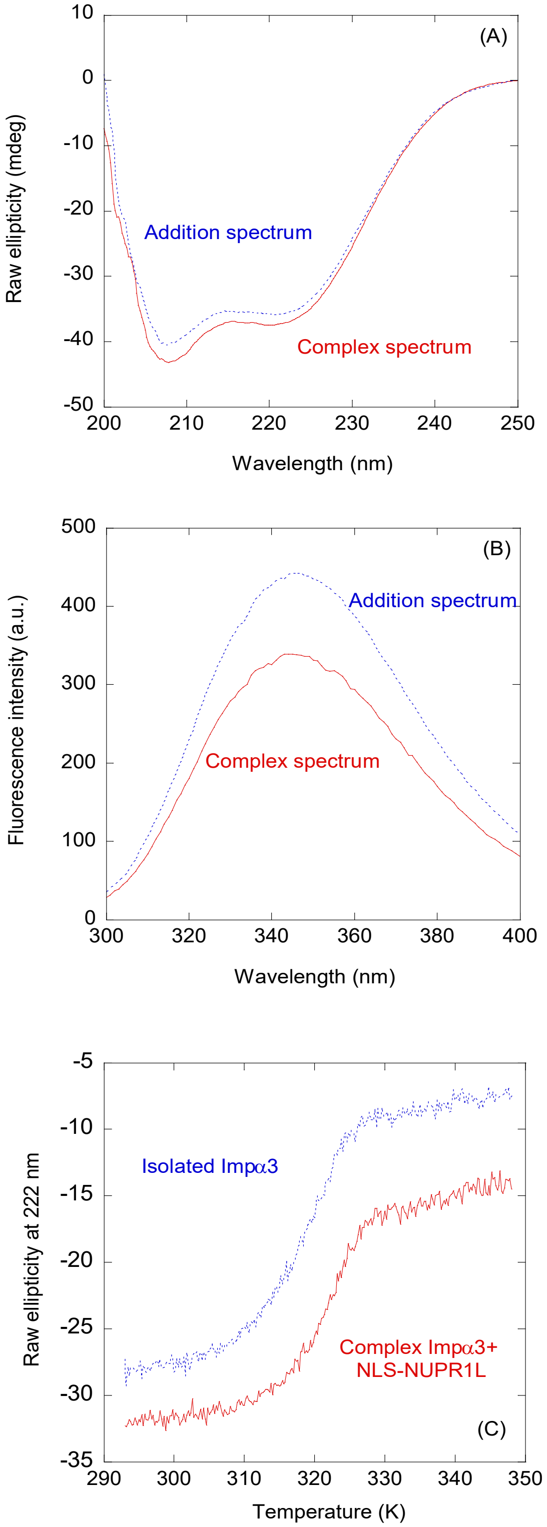

SUPPLEMENTARY TABLE

Table ST1: Chemical shifts (δ, ppm from TSP) of NLSp8L wild-type in aqueous solution (pH 7.2, 283 K)[a]

	NH	Hα	Hβ2	Hβ3	Hγ2	Hγ3	Hδ2	Hδ3	Hε	Hζ
Ac-Arg51		4.32 (-0.09)	1.80							
Thr52	8.19	4.24 (-0.10)	4.07		1.05 (Me)					
Arg53	8.55	4.30 (-0.07)	1.80		1.57					
Arg54	8.54	4.29 (-0.03)	1.80							
Glu55	8.43	4.26 (0.00)	2.07		2.42					
Gln56	8.48	4.32 (0.03)	2.07		2.37					
Ala57	8.31	4.28 (-0.01)	1.37 (Me)							
Leu58	8.17	4.32 (-0.02)	1.63		1.60		0.90 (Me)			
Arg59	8.55	4.35 (-0.09)	1.80							
Thr60	8.31	4.37 (0.08)	4.23		1.21 (Me)					
Asn61 (c)	8.32	4.67 (0.02)	2.65		6.94; 7.59					
Asn61 (t)	8.60	4.70 (0.05)	2.80							
Trp62 (c)	8.23	4.71 (-0.26)	3.06; 3.34		10.12 (NH); 7.22 (2H); 7.52 (7H); 7.15 (5H) 7.20 (6H); 7.69 (4H)					
Trp62 (t)	8.28	4.60 (-0.37)	3.12; 3.41		10.27 (NH); 7.25 (2H); 7.54 (7H); 7.15 (5H) 7.20 (6H), 7.61 (4H)					
Pro63 (c)		4.65 (0.36)	1.95		2.24		3.52; 3.78			
Pro63 (t)		4.50 (0.21)	2.03		2.30		3.70; 3.88			

Ala64 (c)	8.28	4.50 (0.08)	1.37 (Me)		
Ala64 (t)		4.59 (0.17)	1.35 (Me)		
Pro65		4.43 (-0.04)	1.95	2.25	3.64; 3.77
Gly66	8.66	3.98 (0.03)			
Gly67	8.42	3.93 (-0.04)			
His68	8.06	4.63 (-0.02)	3.14	7.17 (C4H); 8.29 (C2H)	
Glu69	8.60	4.23 (-0.04)	1.96	2.25	
Arg70	8.53	4.24 (-0.07)	1.95		
Lys71	8.51	4.25 (-0.09)	1.90		
Val72	8.33	4.08 (0.01)	2.03	0.96 (Me)	
Ala73	8.51	4.28 (-0.03)	1.37 (Me)		
Gln74-am	8.42	4.24 (-0.05)	2.03	2.32	

[a] The (c) and (t) indicate the cis and trans confromations. For the H_α proton column, the value within parenthesis is the conformational shift (δ_{res}-δ_{rc}), where δ_{res} is the chemical shift of such proton for the corresponding residue. The random-coil values for the sequence, δ_{rc}, were obtained from: https://spin.niddk.nih.gov/bax/nmrserver/Poulsen_rc_CS/).
